# Special issue: new horizons in cerebellar research

**DOI:** 10.1186/s40673-017-0076-4

**Published:** 2017-12-29

**Authors:** Adriana B. Conforto, Dennis J. L. G. Schutter

**Affiliations:** 1 Hospital das Clinicas/São Paulo University and Hospital Albert Einstein, São Paulo, Brazil; 2 Donders Institute, Radboud University Nijmegen, Nijmegen, the Netherlands

## Abstract

The feasibility to administer magnetic and electric fields in a non-invasive manner to influence brain areas has attracted scientists interested in studying the neural correlates of normal and pathological forms of behaviour. In particular, the possibility of non-invasive brain stimulation techniques to target the cerebellum has led to a notable rise of research dedicated to unravelling the functional contributions of the cerebellum to motor- and non-motor related behavior. In this issue of *Cerebellum & Ataxias* a series of empirical and review articles provide a state of the art overview of non-invasive brain stimulation of the cerebellum.

## Editorial

The feasibility to administer magnetic and electric fields in a non-invasive manner to influence brain areas has attracted scientists interested in studying the neural correlates of normal and pathological forms of behaviour. In particular, the possibility of non-invasive brain stimulation techniques to target the cerebellum has led to a notable rise of research dedicated to unravelling the functional contributions of the cerebellum to motor- and non-motor related behavior. In this issue of *Cerebellum and Ataxias* a series of empirical and review articles provide a state of the art overview of non-invasive brain stimulation of the cerebellum.

The study by Ben Taib and Manto [1] reports reductions of the inhibitory effect of low-frequency electrical stimulation of the motor cortex (LFSMC) on corticomotor excitability when LFSMC is combined with cathodal direct current stimulation (cDCS) over the cerebellum in anesthesized rats. Corticomotor excitability was evaluated at baseline, after LFSMC alone, and after a combination of LFSMC and cDCS. CDCS was delivered to the left cerebellar hemisphere while LFSMC was administered to the right motor cortex.

Results showed that, similar to the inhibitory effects of low-frequency repetitive transcranial magnetic stimulation, the LFSMC-related decrease in corticomotor excitability was counteracted by cDCS to the cerebellum. This study provides evidence that cerebellar cDCS may be a powerful tool to modify responsiveness of corticomotor excitability.

If cDCS in animals has similar neurophysiological effects to those of cathodal tDCS in humans, and if the effects are not strictly mediated by changes in excitability at the level of the spinal cord, then this approach may have clinical implications in cerebellar patients. For instance, the administration of cathodal tDCS to the cerebellar hemisphere contralateral to the damaged motor cortex in patients might enhance excitability and potentially improve motor symptoms.

In a sham-controlled study by John and colleagues [2] aimed at improving motor performance in a grip force control task, anodal tDCS to the cerebellum was not effective in subjects with cerebellar degeneration nor healthy participants. Among the many possible explanations, task sensitivity as well as task-specificity of tDCS or lack thereof are methodological issues that need to be taken into account for understanding the effects of cerebellar tDCS. This may especially be important in assessing possible clinical benefits of cerebellar tDCS, a field that has gained significant momentum in the last couple of years.

Along this line, Ferrucci and colleagues [3] reviewed effects of cerebellar cathodal or anodal transcranial direct current stimulation (tDCS) in neurological patients with cerebellar injury, including cerebellar ataxias, dystonia, essential tremor and levodopa-induced dyskinesias in Parkinson´s disease. In spite of the limited available evidence, administration of cerebellar anodal tDCS seems to favor cathodal tDCS for improving motor outcomes. As only two studies have yet evaluated the effects of cerebellar cathodal tDCS and six studies assessed the effects of anodal tDCS, more work in this area is warranted.

The review by Tremblay and colleagues [4] explored the potential of non-invasive brain stimulation as a tool to examine cerebellar-M1 interactions in human plasticity. Paired-pulse stimulation protocols, in which an electromagnetic pulse to the primary motor cortex (M1) preceded by an electromagnetic pulse to the cerebellum causes a marked inhibition of corticospinal excitability in comparison to M1 stimulation alone, allow for studying the dentate-thalamo-cortical pathway in vivo. Both repetitive transcranial magnetic (TMS) and electric stimulation (TES) have been applied to the cerebellum to study the effects on dentate-thalamo-cortical output and plasticity in healthy and clinical populations. Their qualitative analysis of twenty-seven studies showed that the effectiveness of paired-pulse stimulation protocols to evoke cerebellum-related plasticity in M1 is subject to a high level of variability. However, sensorimotor protocols designed to lower corticospinal excitability as well as rTMS-TES protocols to lower cerebellar inhibition show reproducible effects in healthy volunteers. The authors discuss these results in light of methodological issues and our current understanding of the physiological mechanisms of cerebello-M1 connectivity.

Even though tDCS to the cerebellum is still in its infancy, the currently available empirical evidence underlines the significant potential of this non-invasive brain stimulation technique in studying cerebellar functions. Further technical and methodological development will undoubtedly contribute to further refinement of tDCS, and the development of clinical applications.
